# Investigation of Cerebral Autoregulation in the Newborn Piglet During Anaesthesia and Surgery

**DOI:** 10.1007/978-1-4939-0620-8_22

**Published:** 2014-03-22

**Authors:** Gemma Bale, Aaron Oliver-Taylor, Igor Fierens, Kevin Broad, Jane Hassell, Go Kawano, Jamshid Rostami, Gennadij Raivich, Robert Sanders, Nicola Robertson, Ilias Tachtsidis

**Affiliations:** 1 Biomedical Optics Research Laboratory, University College London, Malet Place Engineering Building, Gower St., London, WC1E6BT UK; 2 Institute for Women’s Health, University College London, London, UK; 3 Wellcome Centre for Imaging Neuroscience, University College London, London, UK

**Keywords:** Near-infrared spectroscopy, Ultra-low frequencies, Coherence, Neonatal, Histology

## Abstract

The relationship between cerebral autoregulation (CA) and the neurotoxic effects of anaesthesia with and without surgery is investigated. Newborn piglets were randomly assigned to receive either 6 h of anaesthesia (isoflurane) or the same with an additional hour of minor surgery. The effect of the spontaneous changes in mean arterial blood pressure (MABP) on the cerebral haemodynamics (oxy- and deoxy-haemoglobin, HbO_2_ and Hb) was measured using transverse broadband near-infrared spectroscopy (NIRS). A marker for impaired CA, concordance between MABP and intravascular oxygenation (HbD = HbO_2_ − Hb) in the ultra-low frequency domain (0.0018–0.0083 Hz), was assessed using coherence analysis. Presence of CA impairment was not significant but found to increase with surgical exacerbation. The impairment did not correlate with histological outcome (presence of cell death, apoptosis and microglial activation in the brain).

## Introduction

Neonatal exposure to anaesthesia has been associated with apoptotic death of neurons and long-term impairments in cognition in rodents and non-human primates [1, 2]. There is heightened vulnerability during the period of maximal growth, also known as the brain growth spurt period, which occurs perinatally in the human and piglet brain [3]. Research into the neurotoxicity of anaesthetic agents has previously been limited to rodent and primate models which are neurodevelopmentally different to humans; therefore we undertook the current study using a piglet model to gain further insight into the safety of human neonates subjected to anaesthesia.

From this preliminary study, we have found increased cell death and microglial activation in the newborn piglet following 6 h of anaesthesia compared to a naïve control, and that 1 h of minor surgery during the anaesthesia tended to increase injury further. However, the exact physiological mechanism of the injury remains unclear and needs investigation.

It was further observed that the range of mean arterial blood pressure (MABP) changes in the piglets that underwent surgery during anaesthesia was narrower than those that did not. In this paper we hypothesize that an impairment of the cerebral autoregulation (CA), the mechanism that protects the brain by limiting the cerebral blood flow (CBF) variation over a range of arterial blood pressures, may have effect on the histological outcome and will therefore help to explain the histological differences between the two populations (anaesthesia and anaesthesia with surgery).

Many different methods to assess CA impairment through analysis of MABP and markers of CBF using near-infrared (NIR) spectroscopy (NIRS) or transcranial Doppler (TCD) have been reported. The most widely performed is coherence analysis [4]; other methods include transfer function analysis that can assess the severity of the impairment [5]; partial coherence where the measured signals are broken down into smaller epochs and the percentage of epochs with significant coherence is then estimated [6]. Rowley and colleagues employed wavelet cross-correlation (WCC) analysis which can find temporal and frequency components of the CA [7]. Recently non-linear computational models were used to interpret measurements and increase understanding of physiological processes, including the level of CA [8]. The techniques that use frequency analysis typically look at the concordance in specific frequency bands ranging from 0.003 Hz up to 0.1 Hz [5]. The frequency bands relate to different physiological mechanisms so therefore can interpret different clinical features [5].

In this study, CA impairment was assessed by the level of coherence between cerebral intravascular oxygenation (HbD), the difference between oxy- and deoxy-haemoglobin (HbO_2_ and Hb), and MABP. HbD is measured non-invasively using NIRS and reflects CBF if the oxygen saturation (SpO_2_) remains constant. The level of CA impairment for each piglet was compared with the protocol (whether the piglet had anaesthesia or anaesthesia with surgery) and its histological outcome.

## Methods

Experiments were conducted under UK Home Office Guidelines. The study was performed on 16 newborn piglets (less than 24 h old) in the Institute of Neurology, University College London. The piglets were randomized into two groups: (i) anaesthesia (ANA, n = 9) induced with IM midazolam followed by intubation and 2 % isoflurane and iv fentanyl (3–6 μg/kg/h) for 6 h (ii) anaesthesia plus surgery (SUR, n = 7), surgical tracheostomy and bilateral inguinal hernia surgery for 1 h followed by 5 h of anaesthesia. MABP, SpO_2_ and cerebral HbD were measured simultaneously (i) during the anaesthesia and (ii) post-surgery during anaesthesia. The NIRS signals were measured on a broadband NIR spectrometer developed in-house that has been previously used to assess the cerebral function of piglets [9] and humans [10]. The emitter and detector optical fibres were co-linear over the ventromedial/temporal region with a line passing through the brain centre. Intensity spectra between 650 and 980 nm were collected continuously every 1 min (0.0167 Hz) and concentration changes in HbO_2_ and Hb were determined using the UCLn algorithm. The systemic data (MABP and SpO_2_) were measured by intravascular catheterisation (SA Instruments 1025L) and pulse oximetry on the hind hoof (Nonin 8600FO), respectively, at 1 Hz by a data acquisition system (NI USB 6343) and stored on a PC. Animals were sacrificed at 6 h and immunohistochemistry was performed on 9 of the 16 subjects (ANA, n = 5. SUR, n = 4) for TUNEL + (cell death [11]), IBA-1 (microglial activation [12]) and caspase-3 (apoptosis [13]). The histology counts were averaged across the brain for each subject.

## Data Analysis

**Fig. 22.1:**
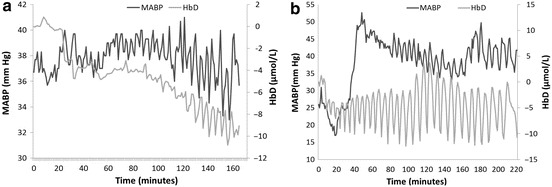
Examples of simultaneous changes in MABP and HbD signals recorded from (**a**) piglet LWP 237 (SUR), Coh_ULF_ = 0.20 with a relatively high coherence as the general trend in MABP is reflected in the HbD and (**b**) piglet LWP 232 (SUR), Coh_ULF_ = 0.06 showing a much lower coherence between the two signals

Spurious data with error values (e.g. −1 for MABP) were considered to be artefact. Artefacts occurring for 2 min or less were removed from the data and interpolated accordingly, artefacts longer than 2 min were truncated. Hence, a single continuous measurement was replaced by a set of continuous artefact-free segments. Figure 22.1 shows two typical examples of the processed signals. Due to problems with the data collection systems, the remaining data lengths range from 19 to 271 min per animal. Ideally only periods in which SpO_2_ varies less than 5 % would be considered to minimize its influence on HbD [4]; however, problems with the data collection system gave insufficient SpO_2_ data for 12 of the subjects. During the experiment, breathing rate and SpO_2_ were well maintained and the SpO_2_ data from the four other subjects was stable within 5 %. Hence, constant SpO_2_ during the procedure has been assumed for all subjects. The 1 Hz systemic data was downsampled to the same time increment as the NIRS data (0.0167 Hz).

Autoregulation impairment was assessed by coherence analysis of the MABP-HbD signals using the Welch method for the estimation of the respective cross-power and auto-power spectral densities [14] implemented in MATLAB (version R2013a, Mathworks). This method involves segmentation of the signals into 19-min epochs (of length equal to the shortest signal) with an overlap of 50 %. The average of the coherence coefficients in the lower end of the ultra-low frequency (ULF) range (0.0018–0.0083 Hz) was then calculated (Coh_ULF_). The upper limit of this frequency range is determined by the Nyquist sampling theorem which avoids aliasing and the lower limit is taken from the window length. The window length is common across all subjects to ensure uniformity and avoid bias from signal length. The coherence coefficients range between 0 and 1 and a coefficient of greater than 0.5 was considered as significant CA impairment [4]. Short lengths of signal were noticed to correlate with higher Coh_ULF_ and so data lengths of less than 99 min were removed to avoid bias. Accordingly three subjects were removed from the study, leaving a remaining group of 13 subjects (ANA, n = 6. SUR, n = 7).

To compare the average Coh_ULF_ values in the two populations (ANA and SUR) a two-tailed Student’s t-test was used. A nominal p-value of less than or equal to 0.05 was considered as statistically significant. The correlation between the histology counts and Coh_ULF_ were valued using R^2^ linear regression and a R^2^ value of greater than 0.5 was considered significant.

## Results

**Fig. 22.2:**
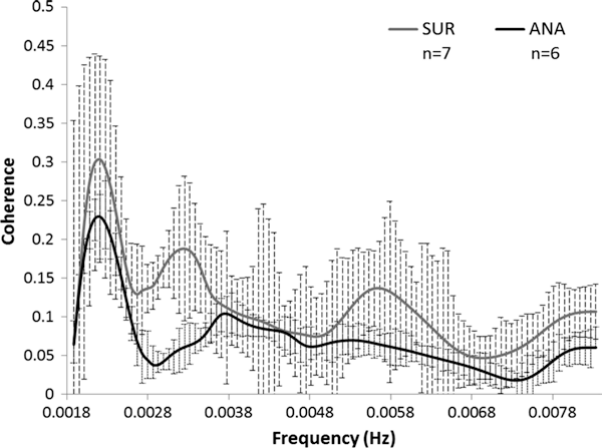
Group average coherence data for anaesthesia (ANA) and anaesthesia plus surgery (SUR) piglets across the ULF range. Significant difference between the groups is observed (p = 0.0498)

**Fig. 22.3:**
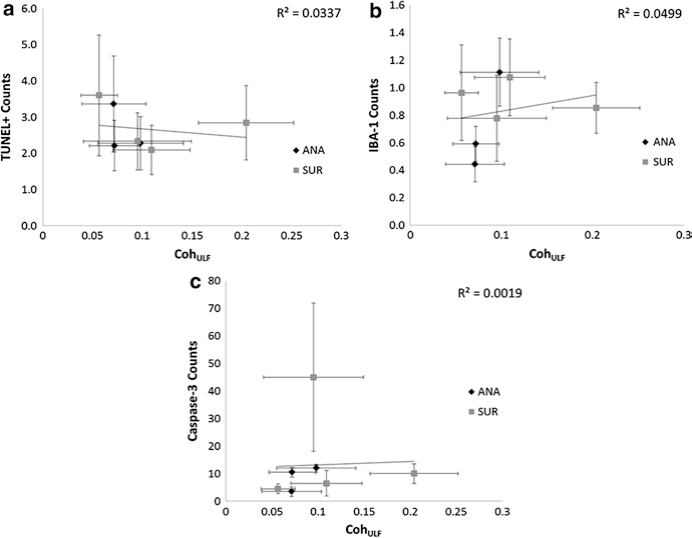
Average Coh_ULF_ for each piglet compared with (**a**) TUNEL + counts, R^2^ = 0.0337, (**b**) IBA-1 counts, R^2^ = 0.0499 and (**c**) caspase-3 counts, R^2^ = 0.0019. Error bars show standard deviation of the mean. The higher the number of counts, the higher the level of (**a**) cell death, (**b**) microglial activation and (**c**) apoptosis, respectively, in the tissue sample

Group Coh_ULF_ scores (mean ± standard deviation) for the ANA group were 0.08 ± 0.02 and for the SUR group 0.12 ± 0.05, and there is a significant difference between the sets (p = 0.050). No piglet showed significant Coh_ULF_ above 0.5 and thus CA was considered to be functioning satisfactorily in all piglets. Figure 22.2 shows the average coherence score for all frequencies across the ULF range for the ANA and SUR groups.The histological counts were plotted against the Coh_ULF_ for each of the seven subjects that had both histological and coherence data (ANA, n = 3. SUR, n = 4), shown in Fig. 22.3. No correlation was observed between any histological test and Coh_ULF_.

## Discussion

No significant levels of Coh_ULF_ between the MABP and HbD were observed in any of the 13 subjects. This suggests that in the ULF range the piglets were autoregulating. The piglets that had surgery with anaesthesia showed a small increase in the level of autoregulation impairment over the piglets that only had anaesthesia. There was no correlation between the histological data and level of CA impairment, suggesting that CA was not involved with the cellular damage. This is reinforced by the low Coh_ULF_ found in all subjects. This suggests that the failure of the CA mechanism was not involved in the apoptotic damage observed after anaesthesia.

These preliminary data suggest that we can eliminate the CA as a potential failing mechanism leading to increased apoptosis and that another physiological mechanism should be investigated. Previous studies [8] have also suggested that CA is not fully developed in newborn piglets and so any CA impairment may be unrelated to the presence of anaesthetic agents. However, the difference between the Coh_ULF_ ANA and SUR groups shows that surgery does stress the CA system, which correlates with the increase in cell death.

This study was limited by several factors. Firstly, the sampling frequency of the data is restricted by the transverse cranial NIRS exposure time and thus restricted the range of frequencies over which the coherence could be observed. Improved data collection methods could reduce the time between exposures and increase the frequency range over which we can test the CA impairment. Additionally, this experiment was not designed to challenge the CA system and thus the range of MABP remained within normal levels so it is possible that the system was not stressed enough to observe CA. The lack of large fluctuations in the MABP results in a lower signal-to-noise ratio which can lower the precision of the Coh_ULF_ measurement [15]. These results are preliminary and more subjects are to be added to the study.
